# Vaccination reduces macrophage infiltration in bronchus-associated lymphoid tissue in pigs infected with a highly virulent *Mycoplasma hyopneumoniae *strain

**DOI:** 10.1186/1746-6148-8-24

**Published:** 2012-03-12

**Authors:** Katleen Vranckx, Dominiek Maes, Silvana B Marchioro, Iris Villarreal, Koen Chiers, Frank Pasmans, Freddy Haesebrouck

**Affiliations:** 1 Department of Pathology, Bacteriology and Avian Diseases, Ghent University, Faculty of Veterinary Medicine, Salisburylaan 133, B-9820 Merelbeke, Belgium; 2 Department of Reproduction, Obstetrics and Herd Health, Ghent University, Faculty of Veterinary Medicine, Salisburylaan 133, B-9820 Merelbeke, Belgium

## Abstract

**Background:**

*Mycoplasma hyopneumoniae *is the causative agent of enzootic pneumonia and is responsible for significant economic losses to the pig industry. To better understand the mode of action of a commercial, adjuvanted, inactivated whole cell vaccine and the influence of diversity on the efficacy of vaccination, we investigated samples from vaccinated and non-vaccinated pigs experimentally infected with either a low (LV) or a highly virulent (HV) *M. hyopneumoniae *strain. Non-vaccinated and sham-infected control groups were included. Lung tissue samples collected at 4 and 8 weeks post infection (PI) were immunohistochemically tested for the presence of T-lymphocytes, B-lymphocytes and macrophages in the bronchus-associated lymphoid tissue (BALT). The number of *M. hyopneumoniae *organisms in bronchoalveolar lavage (BAL) fluid was determined using quantitative PCR at 4 and 8 weeks PI. Serum antibodies against *M. hyopneumoniae *were determined at 0, 2, 4, 6 and 8 weeks PI.

**Results:**

The immunostaining revealed a lower density of macrophages in the BALT of the vaccinated groups compared to the non-vaccinated groups. The highest number of *M. hyopneumoniae *organisms in the BAL fluid was measured at 4 weeks PI for the HV strain and at 8 weeks PI for the LV strain. Vaccination reduced the number of organisms non-significantly, though for the HV strain the reduction was clinically more relevant than for the LV strain. At the level of the individual pigs, a higher lung lesion score was associated with more *M. hyopneumoniae *organisms in the lungs and a higher density of the investigated immune cells in the BALT.

**Conclusions:**

In conclusion, the infiltration of macrophages after infection with *M. hyopneumoniae *is reduced by vaccination. The *M. hyopneumoniae *replication in the lungs is also reduced in vaccinated pigs, though the HV strain is inhibited more than the LV strain.

## Background

*Mycoplasma hyopneumoniae *is the causative agent of enzootic pneumonia (EP) in pigs. This disease is characterized by chronic non-productive coughing and poor growth rate and feed conversion ratio [1]. The disease occurs worldwide and causes significant economic losses to the pig industry [2].

The infection pattern and the severity of the disease in pig herds can be influenced by many factors such as management practices, housing conditions and the virulence of the *M*. *hyopneumoniae *strain [3,4]. Based on protein and genetic studies of field isolates, there appears to be considerable diversity among this species [5,6]. Recently, it has been shown tha^Q1^t within a herd and even within a pig, different strains of *M. hyopneumoniae *can be present [7,8]. In a recent study, it was shown that disease signs aggravated after infection with a highly virulent (HV) strain when pigs had been previously inoculated with a low virulent (LV) strain [9]. The differences in virulence between isolates may be partly due to a differential capacity to provoke an inflammatory response in the host [10].

The diversity between *M. hyopneumoniae *strains also seems to influence the efficacy of vaccination, with better results for some strains [11]. Previous reports have shown that the effect of vaccination may, indeed, vary from herd to herd. In most herds, performance losses due to *M. hyopneumoniae *infection are significantly reduced after vaccination [2]. However, there is no or only limited effect on the transmission of this organism [12,13].

The exact mechanisms of protection after vaccination are not yet fully understood, although both mucosal antibodies and cell-mediated immunity may play a part [14]. The preferential targets of the humoral immune response against *M. hyopneumoniae *are cell-surface proteins, which vary between strains [7,8]. Therefore, antibodies induced by vaccination, might be less effective against infection with certain field strains [15].

Little is known about the effect of different strains on the pig's immune system. Meyns et al. [10] showed that more leukocytes were found in the lung tissue and more IL-1β and TNF-α were detected in broncho-alveolar lavage (BAL) fluid after infection with a HV strain compared to a LV strain. Although the leukocytes were not characterized and only two cytokines were investigated, the HV strain appeared to induce a more severe inflammatory response. The predominant cell types found in the bronchus-associated lymphoid tissue (BALT) after natural infection with *M. hyopneumoniae *are macrophages, but T-lymphocytes, B-lymphocytes and to a lesser extent neutrophils are also present [16]. Macrophages are important in the production of several pro-inflammatory cytokines. These cytokines are essential in the resolution of the infection, but are also responsible for tissue damage in the host [16-18].

In the present study, the infiltration of lung tissue with mononuclear cells after infection of pigs with a highly and low virulent *M. hyopneumoniae *strain was compared and the influence of vaccination on this process was studied.

## Results

### Immunostaining

The results of the scoring of the immunostaining are presented in Table 1. When comparing the effect of strain, no significant differences can be seen between the nvHV and the nvLV group though the values of macrophages, T- and B-lymphocytes were consistently highest in the nvHV group. In the groups infected with the HV strain, it was noticed that the scoring of T-lymphocytes remained high at 8 weeks post infection while for the other groups a substantial reduction in the score was seen. The difference between nvHV, vHV and control at 8 weeks post inoculation had p-values of 0.054 and 0.064 respectively.

**Table 1:** Scoring of the presence of macrophages, T- lymphocytes and B-lymphocytes in bronchus-associated lymphoid tissue (BALT) and the number of *M. hyopneumoniae *organisms (log) in the bronchoalveolar lavage (BAL) fluid^1^

	Score T-lymphocytes (0-3)		Score B-lymphocytes (0-3)		**Score macrophages**^**2**^ (0-3)		**Log of number of *M. hyopneumoniae *organisms in BAL fluid (log qPCR)**^**2**^	
**Weeks PI**	**4**	**8**	**4**	**8**	**4**	**8**	**4**	**8**

**control**	1.0 ± 0.0	0.0 ± 0.0	0.5 ± 0.5	0.5 ± 0.5	0.0 ± 0.0^a, b^	0.3 ± 0.2	-0.76 ± 0.21^a^	-0.69 ± 0.41^a^
**nvLV**	1.2 ± 0.2	0.5 ± 0.5	2.0 ± 0.5	0.8 ± 0.7	0.7 ± 0.5^a, b^	1.3 ± 0.8	1.25 ± 0.74^a, b^	2.41 ± 0.59^b^
**nvHV**	1.4 ± 0.2	1.2 ± 0.3	2.8 ± 0.2	1.6 ± 0.3	1.6 ± 0.9^b^	1.0 ± 0.4	3.44 ± 0.35^b^	1.89 ± 0.71^a, b^
**vLV**	0.8 ± 0.4	0.5 ± 0.2	1.4 ± 0.6	0.9 ± 0.2	0.0 ± 0.0^a^	0.1 ± 0.1	0.97 ± 0.53^a, c^	2.29 ± 0.39^b^
**vHV**	1.0 ± 0.3	1.0 ± 0.2	1.6 ± 0.4	1.5 ± 0.2	0.0 ± 0.0^a^	0.2 ± 0.1	1.96 ± 0.43^b, c^	1.80 ± 0.48^b^

nv non-vaccinated; v vaccinated; LV low virulent challenge strain; HV highly virulent challenge strain

^1 ^Scoring was performed on samples of vaccinated and non-vaccinated pigs at 4 and 8 weeks after endotracheal inoculation with a low or highly virulent *M. hyopneumoniae *strain. A non-vaccinated and non- infected control group was also included.

^2 ^Different lowercase letters correspond to significantly different values between the groups within a column

At 4 weeks PI, the density of macrophages in the BALT of the non-vaccinated groups was higher compared to the vaccinated groups, though this difference was only significant for the HV strain. At 8 weeks, no significant differences were observed for the macrophages though the score in the non-vaccinated groups remained higher than in the vaccinated groups.

### Quantitative PCR

The number of *M. hyopneumoniae *organisms was highly variable between individual animals, even in animals belonging to the same group. This resulted in few significant differences between groups, though in some cases high numerical differences were seen (Table 1). The highest number of *M. hyopneumoniae *organisms was found in the BAL fluid of the nvHV group at four weeks PI (Table 1). For this group, the number of organisms at 8 weeks PI was lower than at 4 weeks PI, but the opposite was found for the nvLV group. These changes were, however, not significant (p-values: 0.076 and 0.237, respectively). The values in the vaccinated groups were numerically lower than their respective non-vaccinated groups both at 4 and 8 weeks PI. The differences for the groups infected with the low virulent strain were much smaller than for the groups infected with the highly virulent strain. This is also reflected in the p-values. The difference between the nvHV and the vHV group at 4 weeks, when the maximum number of organisms was observed for this strain, has a p-value of 0.099. For the LV strain the maximum number of organisms was observed at 8 weeks PI. The difference between the nvLV and the vLV group at 8 weeks PI has a p-value of 0.998. It is therefore likely that the reduction of the organisms of the HV strain seen by vaccination reflects a true difference measured with insufficient power, but that no true reduction is present for the LV strain.

### Serology

The results of the serological testing can be found in Table 2. Before challenge, serum antibodies against *M. hyopneumoniae *were found only in the vaccinated groups. Two weeks after challenge, a rise was seen in the antibody titer of both the vaccinated and non-vaccinated groups, though the rise was more pronounced in the vaccinated groups. At four weeks PI, all challenged groups showed a higher titer. For the vaccinated groups and the nvHV group, the titer remained stable after 4 weeks. Only in the nvLV group, a further rise was seen at 6 weeks post inoculation. At all time points, the titer in the vaccinated groups was significantly higher than the titer in the non-vaccinated groups.

**Table 2:** Number of pigs with serum antibodies against *M. hyopneumoniae *(IDEXX M. hyo Elisa) and average (± SEM) S/P ratio at 0, 2, 4, 6 and 8 weeks post-inoculation (PI) in the different groups

	Weeks PI				
	0	2	4	6	8
**Control**	0/10	0/10	0/10	0/5	0/5
	0.05 ± 0.02^a^	0.00 ± 0.02^a^	0.12 ± 0.05^a^	0.13 ± 0.09^a^	0.13 ± 0.06^a^
**nvLV**	0/20	1/20	14/20	8/10	8/10
	0.12 ± 0.03^a^	0.12 ± 0.04^a^	0.38 ± 0.07^a, b^	0.59 ± 0.12^a, b^	0.59 ± 0.12^a^
**nvHV**	0/20	2/20	20/20	10/10	10/10
	0.15 ± 0.04^a^	0.12 ± 0.04^a^	0.61 ± 0.06^b^	0.68 ± 0.10^b^	0.67 ± 0.14^a^
**vLV**	19/20	19/20	19/10	10/10	10/10
	0.45 ± 0.06^b^	0.84 ± 0.06^b^	1.11 ± 0.08^c^	1.20 ± 0.10^c^	1.24 ± 0.11^b^
**vHV**	17/20	20/20	20/20	20/20	20/20
	0.46 ± 0.05^b^	0.86 ± 0.06^b^	1.14 ± 0.09^c^	1.24 ± 0.09^c^	1.26 ± 0.09^b^

nv non-vaccinated; v vaccinated; LV low virulent challenge strain; HV high virulent challenge strain

Different lowercase letters correspond to significantly different values between the groups at one time point Pigs were vaccinated three weeks before inoculation

### Correlations

Several correlations between the different parameters were significantly positive or negative and the observed associations were different between 4 and 8 weeks post inoculation (Table 3).

**Table 3:** Spearman correlation coefficients between the different parameters investigated in this study^1^

		Log qPC R	T- lymphocytes	B- lymphocytes	Macrophages	IF	Micro LLS	Macro LLS
**Antibody titer**	0	-0.06	-0.06	-0.31	-0.52*	0.17	-0.02	-0.34*
		0.24	-0.09	-0.03	-0.49*	0.17	0.26	0.19
**weeks PI**	2	0.13	0.02	-0.31	-0.53*	0.03	0.01	-0.26
		0.33*	0.19	0.13	-0.37	0.15	0.33*	0.22
	4	0.20	0.02	-0.11	-0.19	0.17	0.25	-0.01
		0.27	0.32	0.22	-0.29	0.01	0.45**	0.20
	6	-	-	-	-	-	-	-
		0.38*	0.57*	0.42	-0.07	0.08	0.41**	0.25
	8	-	-	-	-	-	-	-
		0.43**	0.68**	0.52*	-0.04	0.12	0.46**	0.29
**Log qPCR**			0.33	0.48*	0.63*	0.08	0.62**	0.52**
			0.33	0.36	0.45	0.30*	0.28	0.39*
**T- lymphocytes**				0.40	0.30	0.15	0.24	0.50*
				0.83**	0.37	0.15	0.66**	0.60*
**B- lymphocytes**					0.76**	0.02	0.56**	0.50*
					0.39	0.26	0.55**	0.53*
**Macrophages**						-0.02	0.67**	0.67**
						0.24	0.11	-0.04
**IF**							0.24	0.13
							0.31*	0.44**
**Micro LLS**								0.67**
								0.43**

* *p *< 0.05; ** *p *< 0.01

^1 ^antibody titer (SP ratio of the serology), the scoring of immune cells (T-lymphocytes, B-lymphocytes and macrophages), immunofluorescence staining for *M. hyopneumoniae *(IF) and the microscopic and macroscopic lung lesion score (LLS) at the level of the individual pig at 4 (top) and 8 (bottom) weeks post-infection

## Discussion

In concordance with previous studies, we observed infiltration of macrophages and lymphocytes in BALT after infection with *M. hyopneumoniae *[16,19]. Less macrophages were seen in the BALT of the vaccinated pigs compared to the non-vaccinated animals, although this was only significant for the HV strain. This is the first study to show such effect. Most studies on the mechanisms of vaccination focus on serum antibodies, neutrophils, lymphocytes and cytokine expression [10,20,21]. Okada et al. [22] observed a reduction of lymphocytes, macrophages and neutrophils in the BALF of vaccinated animals compared to non-vaccinated after experimental infection. Thacker et al. [14] reported less TNF-α in vaccinated animals after experimental infection with *M. hyopneumoniae*. As the production of this cytokine is induced in macrophages after contact with *M. hyopneumoniae *[23], this is in concordance with our results.

In the BAL fluid collected from the vaccinated groups, the number of *M. hyopneumoniae *organisms was lower than in the BAL fluid of the non-vaccinated groups. This was also mentioned by Okada et al. [22]. The inhibition of the growth of *M. hyopneumoniae *by this vaccine was, however, more pronounced for the HV compared to the LV strain suggesting that vaccination might be more effective against the HV strain. In previous studies, the onset of disease and the peak of clinical symptoms was approximately two weeks later after inoculation with the LV strain compared to the HV strain, however the entire clinical picture remained milder in pigs infected with the LV strain [9,11]. It is therefore likely that the two time points included in this trial, did not represent the peak of infection for the LV strain. However, this fact alone cannot explain the more pronounced reduction of organisms for the HV strain compared to the LV strain.

An important first step in the innate immune response against *M. hyopneumoniae *is the recognition of mycoplasmal antigens by the TLR2/TLR-6 complex. Though it is not the only pathway involved, the resulting downstream pathway leads to the stimulation of the adaptive immune response [24]. The adaptive immune response is responsible for the elimination of the pathogen. However, for *M. hyopneumoniae *evidence exists that the stimulation of both innate and adaptive immune response is also involved in lung tissue damage [25]. We do not have any knowledge about the overall expression patterns of cell-surface proteins of both strains used in the present study, but it is known that there are differences in the proteome of both strains [26] as well as in the VNTRs (variable number of tandem repeats region) of several cell-surface proteins and hypothetical cell-surface proteins [8]. For one cell-surface protein, the adhesin p97, it has been shown that at least eight pentapeptide tandem repeats in R1 are required to bind porcine cilia [27]. It is therefore not unlikely that different strains have a different binding affinity to host proteins including the TLR2/TLR6 complex and thus activate this complex to a varying degree resulting in a different disease outcome. Therefore the resulting downstream pathways may be more important in the stimulation of the immune response for one strain than for another. As a recent study reported that vaccination may reduce the expression of TLR6 [28], this may result in a more pronounced reduction of tissue damage by strains that rely more on the TLR2/TLR6 complex to stimulate the immune response.

Though it is well known that the outcome of *M. hyopneumoniae *infections can vary considerably between individual animals, even in experimental circumstances, very few efforts have been made to identify the factors influencing this outcome. In the present study, we investigated which parameters were associated with the severity of the disease, determined by the macroscopic and/or microscopic lung lesion score. It became clear that a higher lung lesion score was associated with a higher number of *M. hyopneumoniae *organisms in the BALF and the presence of more immune cells in the BALT. This association was more evident in the non-vaccinated infected groups compared to the vaccinated groups (data not shown). No causal relationship can be deducted from our data, though we suggest the hypothesis that more *M. hyopneumoniae *organisms cause a higher infiltration of immune cells and this causes more tissue damage leading to more severe lung lesions. Meyns et al. [10] observed the same tendencies in an experimental infection study with caesarean-derived colostrums-deprived (CDCD) piglets, though only a small number of animals was included in this study. Ahn et al. [29] found a clinically relevant, though non-significant, association between the concentration of IL-1 in the lung tissue and the microscopic lung lesion score. The current study is the first to suggest a connection between the number of *M. hyopneumoniae*, the induction of immune cells and the severity of the lung lesions at the level of the individual pig. It should be noted that the data from qPCR showed much stronger correlations with the microscopic and macroscopic lung lesions compared to IF suggesting that qPCR is more suitable to investigate the number of organism that IF.

The titer of serum antibodies against *M. hyopneumoniae *was not associated with severity of the lung lesions at 4 weeks post-infection, but a high titer at later time points (6 and 8 weeks PI) was associated with a higher density of immune cells and a higher microscopic lung lesion score at 8 weeks PI. This not only confirms the absence of a link between serum antibody titer and protection against infection [30], but it also suggests that a sustained serum response is related to the continuing presence of clinical signs.

## Conclusions

Although this study was performed on a limited number of samples, some interesting observations were made. Our results suggest that the infiltration of macrophages in BALT after infection with *M. hyopneumoniae *is reduced by vaccination. The growth of *M*. *hyopneumoniae *organisms in the lungs is also reduced in vaccinated pigs, though the HV strain is inhibited to a higher extent than the LV strain. This indicates that future studies should focus on the interaction between macrophages and lymphocytes after infection with different strains and the influence of vaccination on this interaction.

## Methods

### Experimental design

Samples were obtained during a previously described trial [11]. The study was performed after approval of the Ethical Committee for Animal Experiments of the Faculty of Veterinary Medicine, Ghent University. Briefly, 90 piglets from a *M. hyopneumoniae *and PRRSV free farm, were injected at one week of age with either a one-shot commercial, adjuvanted, whole-cell vaccine (v) (Stellamune^® ^One, Pfizer Animal Health) or PBS (nv). At three weeks of age, piglets were weaned and transported to the experimental facilities of the Faculty of Veterinary Medicine, Ghent University, Belgium. After one week, 40 pigs were inoculated with a highly virulent (HV) *M. hyopneumoniae *isolate (F7.2 C), 40 with a low virulent (LV) *M. hyopneumoniae *isolate (F13.10B) and 10 with sterile medium. This resulted in five groups: nvLV, nvHV, vLV, vHV and a non-vaccinated non-infected control group. Half of the piglets in each group were euthanized at 4 weeks post inoculation (PI), the remaining half at 8 weeks PI. The immunofluorescence scoring of *M. hyopneumoniae*, the histopathological scoring (microscopic lung lesion score) and the macroscopic lung lesion score determined in Villarreal et al. [11] can be found in Table 4.

**Table 4:** Immunofluorescence score (IF), microscopic and macroscopic lung lesion score (LLS)^1^

	IF		Microscopic LLS		Macroscopic LLS	
Scores	(0-3)		(0-5)		(0-35)	
**Weeks PI**	**4**	**8**	**4**	**8**	**4**	**8**

**control**	0.00 ± 0.00^a^	0.00 ± 0.00^a^	1.17 ± 0.08^a^	1.17 ± 0.13^a^	0.00 ± 0.00^a^	0.00 ± 0.00^a^
**nvLV**	0.77 ± 0.20^a, b^	1.33 ± 0.20^b, c^	2.90 ± 0.26^b^	2.92 ± 0.15^b^	2.38 ± 1.20^a, b^	1.20 ± 0.30^a^
**nvHV**	1.26 ± 0.27^b^	1.44 ± 0.28^b^	4.04 ± 0.15^c^	3.62 ± 0.15^b^	6.69 ± 1.70^b^	5.60 ± 1.20^b^
**vLV**	0.90 ± 0.21^a, b^	1.33 ± 0.13^b, c^	3.10 ± 0.16^b^	3.41 ± 0.17^b^	0.93 ± 0.90^a^	1.74 ± 0.40^a^
**vHV**	0.80 ± 0.11^a, b^	0.67 ± 0.14^a, c^	3.02 ± 0.09^b^	3.35 ± 0.07^b^	0.11 ± 0.07^a^	1.99 ± 0.60^a^

nv non-vaccinated; v vaccinated; LV low virulent challenge strain; HV highly virulent challenge strain

^1 ^Scoring was performed in vaccinated and non-vaccinated pigs at 4 and 8 weeks after endotracheal inoculation with a low or highly virulent *M*. *hyopneumoniae *strain. A non-vaccinated and non- infected control group was also included. The parameters were determined in the study of Villarreal et al. (2011) [11] and included in the statistical analysis of the present study.

### Immunostaining

For each group, lung tissue samples from five randomly selected pigs were investigated at 4 and 8 weeks PI, except for the control group, from which only two pigs were selected. Formalin fixed and paraffin embedded lung samples collected for histopathological examination for the study of Villarreal et al. [11] were examined and if available, samples containing BALT were selected for immunostaining. The primary antibodies used were polycolonal rabbit antibodies against human T-lymphocytes CD3 (Dako A0452, Heverlee, Belgium), and B-lymphocytes CD20 (Neo Markers RB-9013-P, Duiven, the Netherlands) and monoclonal mouse antibodies against macrophages MAC387 (Serotec MAC387, Oxford, UK) [31]. The immunostaining was based on Breugelmans et al. [32]. Briefly, antigen retrieval was performed by incubating the slides in citrate buffer, microwave heating, and cooling during 30 min at 4°C. Non-specific binding was prevented by incubation of the slides with 30% normal rabbit serum in PBS during 30 min at room temperature. Samples were then incubated for 1 hour at room temperature with the optimal dilutions of the antibodies in PBS (CD20 and MAC387) or tris-buffered saline (CD3). After washing, the endogenous peroxidase activity was blocked by incubation at room temperature in 3% H2O2 for 5 min. After washing with the buffer solution, the sections were incubated with a labelled polymer-HRP goat anti-rabbit (CD3 and CD20) or goat anti-mouse (MAC387) for 30 min at room temperature. The peroxidase activity was revealed by using diaminobenzidine (Dako, Denmark) as chromogen after washing of the sections. The slides were counterstained with hematoxylin for 10 s. After dehydration, the slides were mounted using DPX mountant (VWR Int., Lutterworth, Leicestershire, UK).

Ten images were taken per sample by standard light microscopy at a magnification of 40× (Leitz, New York, USA), covering the whole surface area of the slide. A visual score according to the density of stained cells in the BALT was assigned ranging from 0 (no to a few stained cells) to 3 (almost all cells in BALT stained). All images were scored by the same person and this person was blinded to the groups.

### Quantitative PCR

The number of *M. hyopneumoniae *organisms in the lung was estimated by performing quantitative PCR (qPCR) on the BAL fluid as described previously [33]. Briefly, qPCR was performed with primers 5' GTCAAAGTCAAAGTCAGCAAAC 3'and 5' AGCTGTTCAAATGCTTGTCC 3' using iQ SYBR Green Supermix (Bio-Rad, Eke Nazareth, Belgium) in the CFX96 real-time PCR detection system (Bio-Rad). To convert the threshold values to the number of organisms, a tenfold dilution series of *M. hyopneumoniae *DNA was made in PBS.

### Serology

At 0, 2, 4, 6 and 8 weeks PI, blood samples were taken from all pigs and tested for the presence of antibodies against *M. hyopneumoniae *in the serum using the IDEXX M. hyo. ELISA (IDEXX, Hoofddorp, The Netherlands). The serum antibody titer is estimated by the sample-to-positive (SP) ratios calculated from the optical density (OD)-values: (OD_sample _- average OD_neg control_)/(average OD_pos control _- average OD_neg control_) [34].

### Statistical analysis

One-way analysis of variance (ANOVA) was used to analyze the results from the scoring of immunostaining, qPCR and the serum antibody titer between groups at the same time point. When the homogeneity of variances test was passed, Bonferroni was used as post hoc test, when it failed, Dunnett T3 was used. Data from qPCR was transformed to the logarithm prior to analysis to normalize the data. Correlations between macroscopic lung lesion score, microscopic lung lesion score (histopathology), IF, immunostaining, log qPCR and serum antibody titer on the level of the individual pig were determined by calculating the Spearman's rho correlation score (ρ) both for the data 4 and 8 weeks PI. Correlations were also calculated for these three parameters and the antibody titer at and before euthanasia for both 4 and 8 weeks PI. The statistical results were considered significant when p-values were ≤ 0.05. All analyses were performed with SPSS 17.0 for Windows (SPSS inc. Illinois, USA).

## Competing interests

The authors declare that they have no competing interests.

## Authors' contributions

KV, DM, IV, FP and FH participated in the conception and design of this study. KC contributed in the conception and design of the immunostaining. KV and IV participated in the acquisition of data. KV and SBM participated in the analysis and interpretation of data. KV, DM and FH drafted the manuscript. All authors read and approved the final manuscript.
